# A1 TARGETING IL-4 AND IL-13 TO TREAT CROHN’S DISEASE-ASSOCIATED INTESTINAL FIBROSIS IN SHIP DEFICIENT MICE

**DOI:** 10.1093/jcag/gwac036.001

**Published:** 2023-03-07

**Authors:** K Safari, S Menzies, H Jung, L Sly

**Affiliations:** Pediatrics, BCCHRI; Pediatrics, BCCHRI; Pediatrics, BCCHRI; Pediatrics, University of British Columbia, Vancouver, Canada

## Abstract

**Background:**

Crohn’s disease (CD) is an immune-mediated disease characterized by chronic, relapsing and remitting, or progressive inflammation of the digestive tract. One in 3 people with CD will develop intestinal fibrosis requiring surgery within 10 years of diagnosis. Biological therapy is effective at reducing inflammation in CD and early and aggressive treatment with biologics will reduce the incidence of fibrosis. However, in children, for whom a step-up approach to therapy is used, and populations that are refractory to biological therapy; fibrosis remains a serious concern. Treatments for intestinal fibrosis are needed as currently, there are no treatment that target intestinal fibrosis directly.

Mice deficient in the Src homology 2 domain-containing inositolpolyphosphate 5’-phosphatase (SHIP-/-) develop spontaneous CD-like ileal inflammation and fibrosis. Fibrosis is dependent on PI3Kp110d activity, leading to induction of the enzyme arginase I (argI). Genetic ablation or pharmacologic inhibition of PI3Kp110δ or inhibition of arginase activity block the development of intestinal fibrosis in SHIP-/- mice. IL-4 and IL-13 can activate PI3Kp110δ and IL-4 concentrations are elevated systemically in SHIP-/- mice. Thus, we hypothesize that SHIP-/- mice develop CD-like intestinal fibrosis due to increased IL-4 and/or IL-13 signalling.

**Purpose:**

**Aim 1:** To determine whether genetic ablation of IL-4 prevents ileal fibrosis in SHIP-/- mice

**Aim 2:** To determine whether a blocking antibody directed against IL-4 or IL-13

**Method:**

SHIP-/- mice were crossed with IL-4 deficient mice (IL-4-/-) to generate wild type, SHIP+/+IL-4-/- SHIP-/-IL-4+/+, and SHIP-/-IL4-/- mice. SHIP-/- mice (6-week-old) were treated with an anti-IL-4 antibody or an anti-IL-13 antibody, IgG isotype control, or PBS (injection control), for 2 weeks. Ileal fibrosis was assessed in SHIP-/- mice comparing mice treated with anti-IL-4 antibody or anti-IL-13 antibody to IgG controls. Measurements of ileal fibrosis included gross and histopathology, muscle thickening, and collagen accumulation (Masson’s trichrome staining for fibrosis).

**Result(s):**

SHIP-/-IL-4-/- mice did not have less ileal fibrosis than their SHIP-/- littermates and blocking IL-4 in SHIP-/- mice did not reduce ileal fibrosis. However, blocking IL-13 in SHIP-/- mice reduced ileal fibrosis.

**Conclusion(s):**

Blocking IL-4 genetically or with a blocking antibody is insufficient to reduce ileal fibrosis in SHIP-/- mice. Blocking IL-13 with a blocking antibody is sufficient to block the development of ileal fibrosis in SHIP-/- mice. In future experiments, we will cross SHIP-/- mice with IL-13-/- mice to determine whether targeting IL-13 genetically also reduces fibrosis in SHIP-/- mice. We will also determine which receptor IL-13 is signalling through to indue fibrotic pathology in SHIP deficient mice. Our data suggest that an antibody-based biologic targeting IL-13 may be an effective strategy to prevent fibrosis in people with Crohn’s disease.

**Disclosure of Interest:**

None Declared

